# *Erechtites hieracifolia*: an invasive plant species in peatland habitats of southeastern Poland (Central Europe)

**DOI:** 10.3389/fpls.2025.1615073

**Published:** 2025-09-10

**Authors:** Agata Stadnicka–Futoma, Małgorzata Jaźwa, Konrad Kata, Ewelina Klichowska

**Affiliations:** ^1^ Department of Soil Science, Environmental Chemistry and Hydrology, Institute of Agricultural Sciences, Environment Management and Protection, Faculty of Technology and Life Sciences, University of Rzeszów, Rzeszów, Poland; ^2^ Institute of Biology, Faculty of Natural Sciences and Technology, Department of Botany and Biological Monitoring, University of Opole, Opole, Poland; ^3^ Independent Researcher, Kolbuszowa, Poland; ^4^ Institute of Botany, Faculty of Biology, Jagiellonian University, Kraków, Poland

**Keywords:** *Erechtites hieracifolia*, invasive plant, dynamic population, peatland, endangered habitat

## Abstract

**Introduction:**

*Erechtites hieracifolia* is an invasive plant species increasingly colonizing peatland plant communities in Central Europe. Invasive plant species are a growing global concern, as they colonize a wide range of habitats, contributing to biodiversity loss. Anthropogenic activity and climate change intensify this process. Mires are among the most vulnerable ecosystems, as lowering groundwater levels and habitat changes facilitate the penetration of invasive species. The aim of this study was to analyze the floristic composition of peatland plant communities with the presence of *E. hieracifolia*, including an assessment of potential differences between plots with and without the species. In addition, changes in population size over a 3-year period were evaluated, and new localities of *E. hieracifolia* in Poland were identified.

**Methods:**

In 2019, a total of 60 phytosociological relevés were taken in 12 peatland patches (six with the presence of *E. hieracifolia* and six without it). The study was repeated in 2022 on the same patches. A syntaxonomic classification of the plant communities was established. The PERMANOVA method was used to analyze differences in species composition between patches with and without *E. hieracifolia*. Biodiversity indices (Shannon–Wiener and Simpson) were also compared between these two groups.

**Results:**

*E. hieracifolia* most frequently occurred in raised bogs with low groundwater levels within the *Sphagno recurvi–Eriophoretum vaginati* association. Maximum species cover was recorded in patch 11 (25%). Within 3 years, the number of patches with *E. hieracifolia* increased from six to eight. In some of these patches, an increase in both the species cover and range was observed, indicating ongoing expansion. Statistical analysis revealed significant differences in species composition between patches with and without the presence of *E. hieracifolia*. A clear association of the species with patches characterized by a more abundant occurrence of *Eriophorum vaginatum* tussocks was also observed. The Shannon–Wiener and Simpson diversity indices showed slightly lower species diversity in communities with *E. hieracifolia*, suggesting that habitat changes associated with peatland drying favor its colonization.

**Discussion/Conclusions:**

The results indicate that *E. hieracifolia* preferentially colonizes degraded habitats characterized by reduced biodiversity. These findings suggest that *E. hieracifolia* may act as an indicator of peatland degradation, with its expansion facilitated by hydrological changes. The study highlights the need for continuous monitoring of invasive species in peatland ecosystems, as their spread may further accelerate biodiversity loss in these vulnerable habitats.

## 1 Introduction

The spread of invasive alien species in the plant world is a current issue, especially in the era of climate change. Humans are the main vector of diasporas, but global warming can enable species to migrate and occupy new locations (Mashhadi and Radosevich, 2004). Invasive plants most often become a threat to biodiversity. They displace native species and reduce species richness by changing habitat conditions. Approximately 50% of alien plant species can be considered economically harmful (Richardson et al., 2000). This problem refers to all countries in the world, and this is also common in Poland. Approximately 40% are species of foreign origin, of which approximately 3% are invasive plants. The greatest threat seems to be species occupying natural habitats (Tokarska-Guzik et al., 2012). Peatlands are among such habitats exposed to the invasion of alien species.

Mires are among the most valuable and at the same time most endangered ecosystems in the world. It is estimated that they occupy approximately 3% of the land surface (Xu et al., 2018). Among other environmental functions, peatlands can reduce the effects of climate change because well-preserved ecosystems of this type store carbon (Bragg and Lindsay, 2003). However, it should be noted that these areas may also become sources of carbon dioxide emissions, as drainage leads to the oxygen intrusion and aerobic decomposition of organic matter, thereby disrupting peat accumulation and releasing greenhouse gases (Pępkowska-Król and Wilk, 2020). As natural retention reservoirs, they store large amounts of water, simultaneously preventing floods and mitigating droughts and the effects of water deficiency. Processes occurring in peatland, such as evaporation and transpiration, cause a local lowering of temperature and an increase in air humidity (Bragg and Lindsay, 2003). They also have the ability to purify water, e.g., from nitrogen and phosphorus, which are accumulated in peat. This role is particularly important when the peatland is located near agricultural land (Pępkowska-Król and Wilk, 2020). One of the important functions is also the preservation of biodiversity (Bragg and Lindsay, 2003). Peatlands are a habitat for rare and endangered plant species. Therefore, the protection of peatlands is of great importance. In many countries, they are protected in the framework of the Council Directive (1992).

During the development of agriculture, European peatland bogs were extensively drained and subsequently exploited. Currently, climate change—rising average temperatures, more frequent heat waves, and prolonged droughts—is contributing to the degradation of wetland habitats. This leads to increased evaporation and transpiration (Lamentowicz and Konczal, 2016), which in turn accelerates the decline of groundwater level, a key factor in the destabilization of peatland hydrology. As a result of drainage and long-term hydrological disturbance, secondary succession initiates on dried-out peatlands. One of the characteristic stages of this process is the establishment of woody vegetation, including trees and shrubs. These species not only intensify evapotranspiration through their biomass and leaf surface but also actively extract water from the substrate, further reducing the groundwater level. Moreover, this modifies light availability, changing the composition and diversity of lower vegetation layers. The penetration of the shrub root system into the catotelm simplifies oxygen transfer (Bragg and Lindsay, 2003), which promotes aerobic decomposition. This leads to accelerated peat mineralization and long-term peat loss (Okuszko et al., 2011). The ecological consequence of these transformations includes the degeneration of native phytocoenoses and a progressive decline in biodiversity. At the same time, disturbed peatlands become more susceptible to colonization by expansive and invasive plant species. Although peatlands are generally resistant to biological invasion due to specific hydrological and chemical conditions, these natural barriers are weaker under disturbance (Zedler and Kercher, 2004). Under such conditions, even some native species—such as *Molinia caerulea* (Bociąg et al., 2017)—may spread aggressively. Invasive alien plants also pose a threat. One of the most aggressive neophytes, *Spiraea tomentosa*, is capable of forming dense and extensive stands, which enhance evapotranspiration and outcompete native flora. Other less impactful species include *Oxycoccus macrocarpus* or *Mimulus guttatus* (Dajdok and Pawlaczyk, 2009), although their ecological roles may vary regionally. A more recent arrival is *Erechtites hieracifolia*, which until recently has been observed mainly in the early stages of invasion, particularly in anthropogenically disturbed areas such as clear-cuts. However, its potential to colonize open peatland areas raises concern. Given the cumulative impact of vegetation changes and groundwater level reduction, it is crucial to carefully study and monitor the behavior of this species, as well as wider trends, to protect the ecological integrity of peatland ecosystems.

The aims of this study were 1) to analyze the floristic composition of peatland communities with and without *E. hieracifolia*, taking into account aspects of population dynamics assessed based on changes in species cover; 2) to show differences in species cover between the analyzed groups (with and without *E. hieracifolia*) and to create a database for future monitoring of its occurrence; and 3) to identify new sites of this species in southeastern Poland. We hypothesized that *E. hieracifolia* preferentially colonizes ecologically degraded habitats characterized by reduced species diversity.

## 2 Materials and methods

### 2.1 Study species

**Figure 1:**
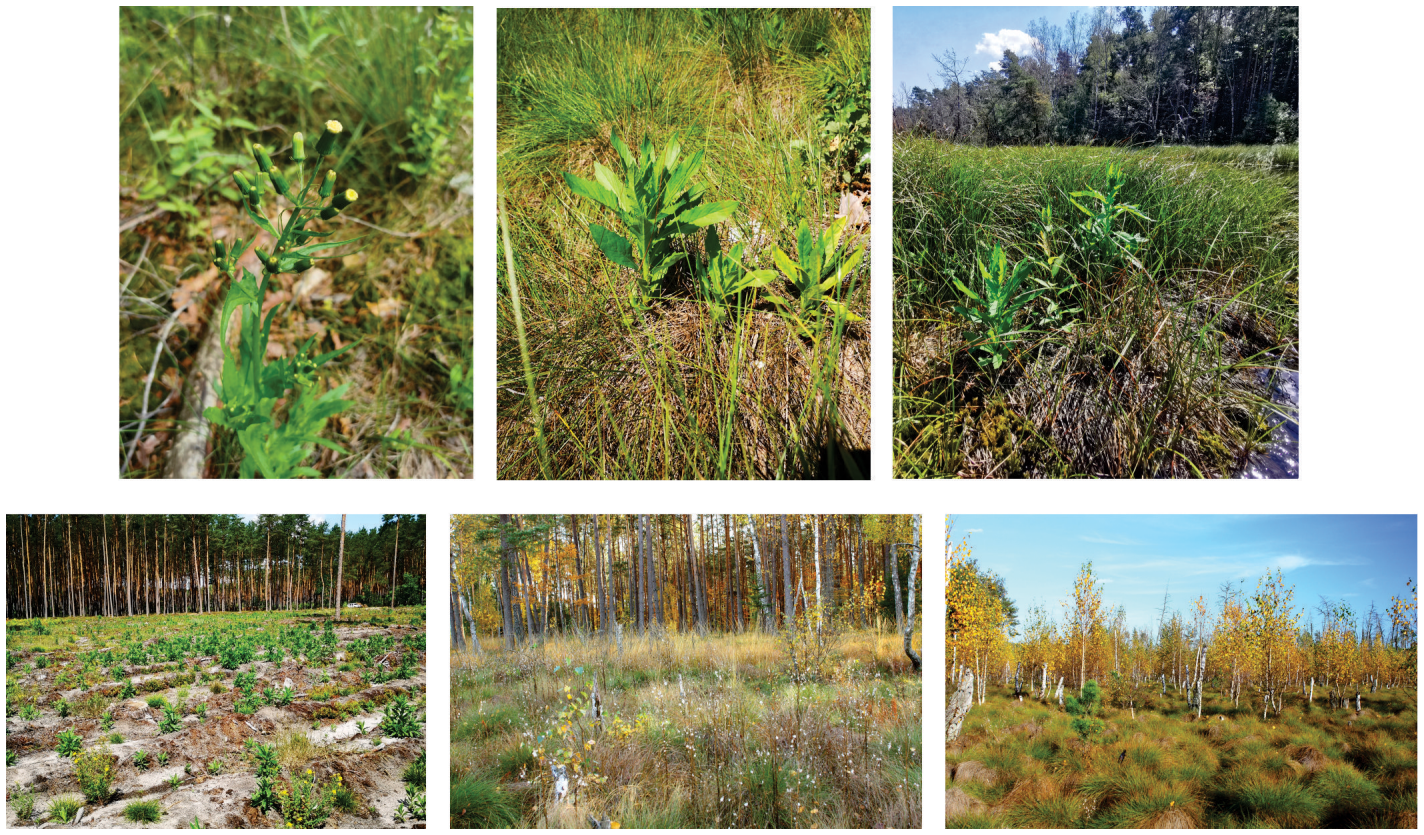
*Erechtites hieraciifolia* and its habitats: from the top left – generative individual; few individuals on a clump of *Eriophorum vaginatum*; few individuals in a floating peat mat in Bagno Przecławskie reserve; from the bottom left – a population in a clear-cut area with massive occurrence as a spreading center; the largest population on the peat bog; Sphagno *recurvi–Eriophoretum* vaginati association as the most common habitat of the species (photos by A. Stadnicka-Futoma and K. Kata).

*E. hieracifolia* (**Figure 1**) is a fast-growing annual plant belonging to the Asteraceae family, reaching a height of 50 to 200 cm. The lanceolate-oblong leaves are short-petioled or sessile, up to 20 cm long and up to 8 cm wide. The flowers are grouped in capitula with pale yellow florets. A single individual produces an average of 253 capitula, arranged in a corymb. The plant flowers from midsummer through early autumn before succumbing to frost. Approximately 130 achenes are produced from each capitulum, resulting in enormous seed output (Rutkowski, 2004; Csiszár, 2006; Darbyshire et al., 2012; Koczywąs et al., 2012; Tichý et al., 2023). Its seeds are released with a downy pappus, allowing them to be carried over long distances, a trait typical of pioneer species (Darbyshire et al., 2012). *E. hieracifolia* is an r-strategy species, characterized by prolific achene production and the presence of a pappus that facilitates long-distance wind dispersal. This effective mechanism, often supported by various dispersal vectors, enables the species to rapidly colonize newly available habitats (Darbyshire et al., 2012; Grime, 1977; Krahulec and Hadinec, 2011). Studies have indicated that *E. hieracifolia* disperses primarily via wind. However, Marciniuk et al. (2019) and Orlov and Iakushenko (2011) reported that vehicles can also transport seeds along roads. Local seed transfer may also occur via forestry machinery used for logging operations (Górski et al., 2003; Von der Lippe and Kowarik, 2007; Bohdan and Sulej, 2020; Zaniewski et al., 2020). Under favorable conditions, it is capable of producing multiple generations within a single growing season (plants.ces.ncsu.edu).

*E. hieracifolia* is a highly ecologically flexible species—it grows in habitats with varying moisture levels, including both wet and dry sites, and even in gravelly or sandy soils. It tolerates both fresh and temporarily flooded conditions, thriving along riverbanks, ditches, low-lying areas, and wetland edges, as well as on rocky and gravelly substrates. It tolerates a wide range of pH—from acidic (pH ~ 4.5) to alkaline (pH ~ 8)—and can grow in soils with varying nutrient availability and salinity (Darbyshire et al., 2012).

**Figure 2:**
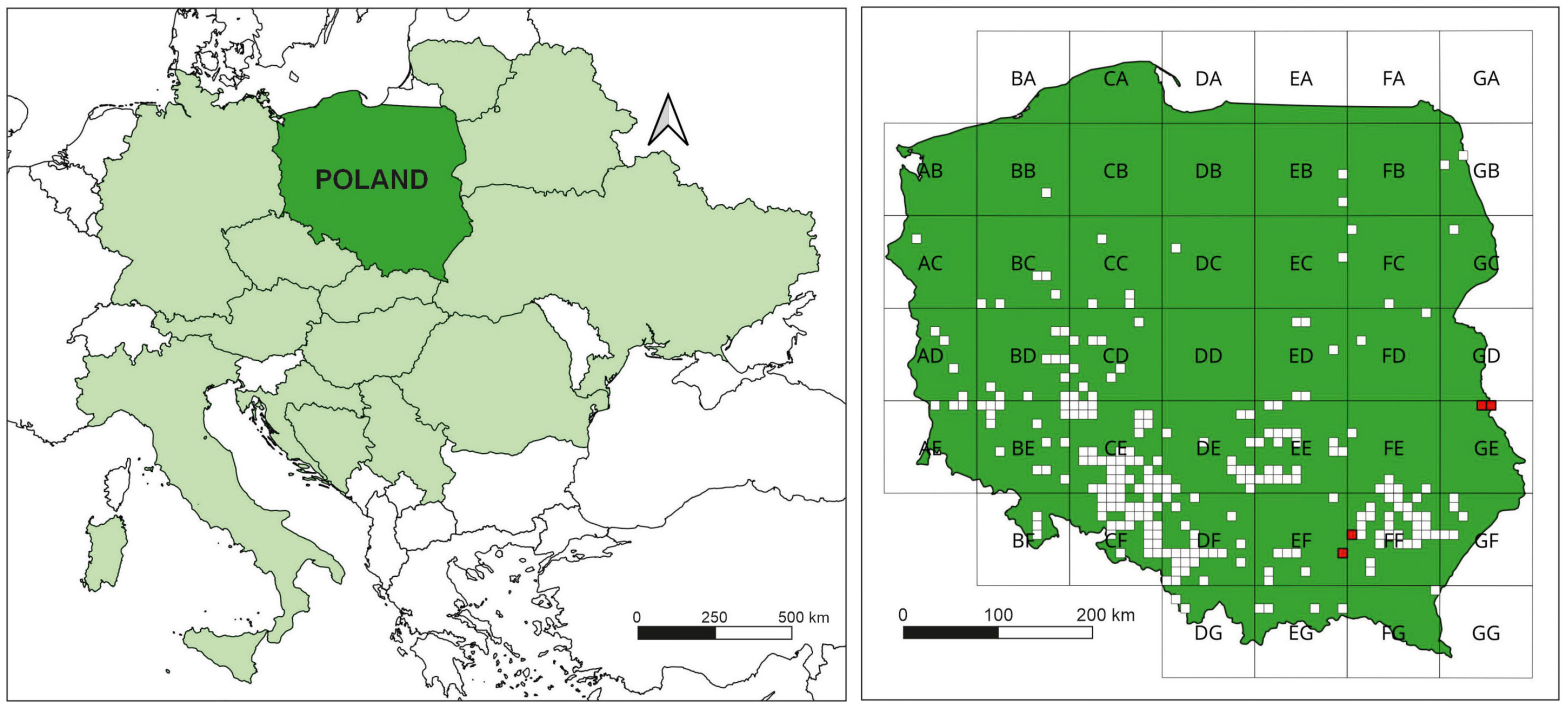
Distribution of *Erechtites hieraciifolia*: left – in Central Europe; right – in Poland (in ATPOL grid squares: white squares – old localities, red squares – new localities; own work based on ATPOL grid squares; Zając and Zając, 2001).

*E. hieracifolia* is native to North and South America (Darbyshire et al., 2012). In Europe, the species was first recorded near Zagreb, Croatia, in 1876 (Dvořáková, 2004). The next records came from Austria, Belarus, Bosnia-Herzegovina, the Czech Republic, Germany, Hungary, Italy, Lithuania, Serbia, Slovakia, Slovenia, Romania, and Ukraine (Klotz and Schuhwerk, 2009; Hadinec and Lustyk, 2011; Krahulec and Hadinec, 2011; Orlov and Iakushenko, 2011; Gudžinskas and Taura, 2020; Dubovik et al., 2021; Mosyakin and Mosyakin, 2021; Trotta et al., 2023; Kermavnar and Kutnar, 2024) (**Figure 2-left**). It appeared in Poland in 1902 in Lower Silesia (Schube, 1903). The detailed history of the species’ spread is presented in the study by Zaniewski et al. (2020). The distribution map has been revised with new sites (**Figure 2B-right**). *E. hieracifolia* has been recognized as a settled kenophyte capable of colonizing anthropogenic, semi-natural, and natural habitats (Tokarska-Guzik et al., 2012).

In Europe, it most often colonizes degraded areas in forests, mainly pine and mixed forests and acidophilous oak forests, as well as oak-hornbeam habitats. It particularly chooses clearings, clear-cuts, forest plantations, windfalls, and forest roadsides or their edges (e.g., Górski et al., 2003; Chmura, 2004; Tokarska-Guzik et al., 2009; Krawczyk, 2010; Hadinec and Lustyk, 2011; Krahulec and Hadinec, 2011; Orlov and Iakushenko, 2011; Bartoszek, 2019; Marciniuk et al., 2019; Bohdan and Sulej, 2020). Less frequently, it is in ruderal habitats such as the sides of asphalt roads, including motorways (e.g., Tokarska-Guzik et al., 2009; Krahulec and Hadinec, 2011; Orlov and Iakushenko, 2011). Nowak (2014) found it in gardens where earthworks were carried out and the structure of the substrate was disturbed. It can often occupy various habitats after fires (Orlov and Iakushenko, 2011; Zaniewski et al., 2020). Less frequently, it is found in wetland communities, e.g., reed beds such as *Phragmitetum australis* (Cameron and de Lange, 2002; Spałek, 2015) and peatland (e.g., Górski et al., 2003; Krawczyk, 2010; Orlov and Iakushenko, 2011). It also inhabits the edges of oxbow lakes, wet pastures, and embankments (Krawczyk, 2010).

New locations and occupied habitats are the subject of numerous studies on the species in Poland and other European countries (e.g., Górski et al., 2003; Klotz and Schuhwerk, 2009; Kocián and Kocián, 2009; Hadinec and Lustyk, 2011; Orlov and Iakushenko, 2011; Wolanin, 2014; Kolomiychuk et al., 2019; Marciniuk et al., 2019; Gudžinskas and Taura, 2020; Trotta et al., 2023; Wołkowycki and Wołkowycki, 2023). The biology of the Canadian populations of the species and its distribution in Canada and economic importance were described in detail in the work by Darbyshire et al. (2012). Celka et al. (2017) investigated the morphological variation of this species, comparing Polish and Ukrainian materials. The population dynamics within several habitats after a fire were described by Zaniewski et al. (2020). Among them, the data come from the Długie Bagno peat bog. So far, the rate of change in species contribution to the plant community has not been studied within degraded peat bogs.

### 2.2 Study site and methodology of vegetation surveys

The main research was conducted in southeastern Poland in the Łęczyńsko-Włodawskie Lakeland mesoregion, which is part of the West Polesia macroregion (GE ATPOL square) (**Figure 2- right**). This area is characterized by vast plains formed by periglacial and fluvial processes, interspersed with chalk and moraine hills. The dominant soils are podzolic and rusty soils developed on sandy substrates. Rendzina soils are also present, along with gley and peat soil associated with river valleys and depressions (Dobrowolski and Chabudziński, 2021). Pine and mixed forests dominate this area. The presence of impermeable chalk marls contributes to the formation of numerous lakes and wetland areas (Woźniak et al., 2021). The described region is located within the Natura 2000 area Lasy Sobiborskie PLH060043 and the Sobibór Landscape Park, and it is characterized by unique natural values, including the significant presence of peatland communities. Active raised bogs (7110) and degraded raised bogs still capable of natural regeneration (7120) dominate. Transition mires and quaking bogs (7140), developing primarily around lakes, also occupy a significant area (SDF, 2025). The conservation status of these habitats ranges from favorable to unfavorable–bad. The main threats to these habitats are periodic droughts and the impact of drainage ditches, which lower the groundwater level and accelerate secondary succession. In response to these threats, active conservation measures were initiated in 2019, including the removal of trees and tree saplings.

**Figure 3:**
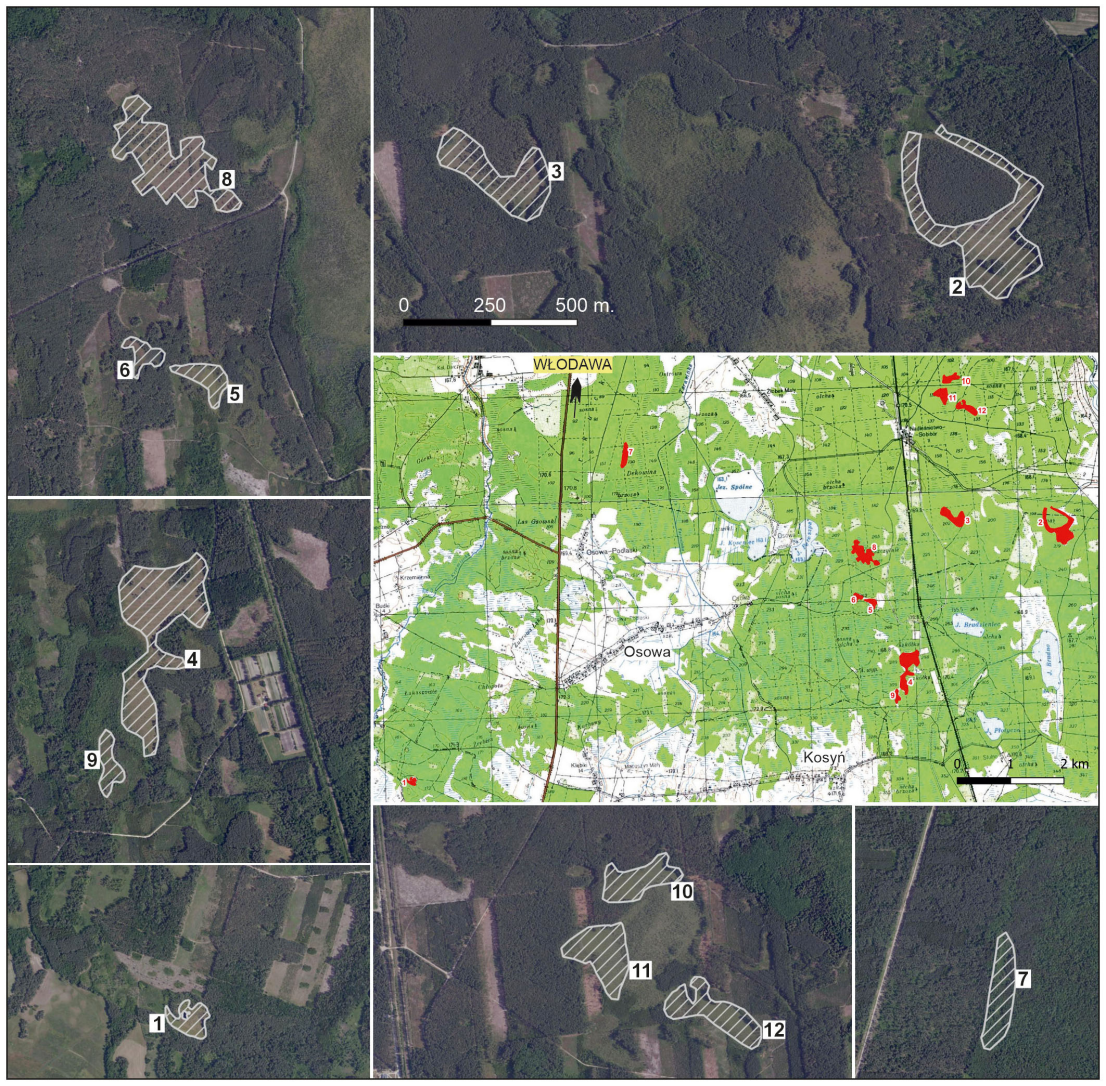
Distribution of patches in the study area: general map with all patches on topographic map as a background; larger-scale patches on orthophotomap as a background.

Twelve peatland patches were randomly selected (including all where *E. hieracifolia* was found) (**Figure 3**). These patches were often heterogeneous, but with a dominant plant community type. Patch boundaries were determined in the field using a GPS receiver by marking boundary points, which were then connected and visualized in QGIS. General information about individual patches is provided in **Table 1** and detailed in **Supplementary Table S1**. Sixty phytosociological relevés (1 – 60) were taken in 2019. In 2022, relevés were repeated in the same locations (1A – 60A).

**Table 1:** Summary of information on the examined patches.

Number of patches	Surface of patches (ha)	Number of phytosociological relevés	Habitat code
1	1.70	58–60	7140
2	15.05	13–17, 42–44	7110
3	8.05	18–21	7110
4	13.91	22, 23, 48–50, 55–57	7110
5	1.96	24, 25	7140
6	1.30	26, 27	7140
7	4.19	39–41	7140
8	10.38	45–47, 51	7110
9	1.58	52–54	7140
10	3.61	1–3, 33–35	7110
11	4.88	4–12	7120
12	4.71	28–32, 36–38	7110
13	–	61	7110
14	–	62	7110

Additionally, newly discovered localities of the species in 2022 were also considered (phytosociological relevés 61 and 62). *E. hieracifolia* was found in the Bagno Przecławskie and Torfy Nature reserves. The research was carried out as part of a field survey, during which an inventory was conducted for the project focused on protecting habitats and species of non-forest areas dependent on water, carried out on behalf of the Regional Directorate for Environmental Protection in Lublin and during research for the Protection Plans commissioned by the Regional Directorate for Environmental Protection in Rzeszów. The nature reserves are located within the Tarnów Plateau mesoregion, which is part of the Sandomierz Basin macroregion (EF and FF ATPOL squares) (**Figure 2-right**). Moraine and denudation plains dominate, often diversified by inland dunes. A mosaic of brown soils, leached brown soils, and podzolic soils has developed on silty parent material (Łanczont and Chabudziński, 2021). Similarly, coniferous and mixed forests have a significant share in this area, with small fragments of peatlands within them.

In total, 122 phytosociological relevés were conducted. The Braun-Blanquet method (Braun-Blanquet, 1964) was used. The plot of each relevé was 25 m^2^ (5 × 5 m). Phytosociological relevés were compiled into a table (**Supplementary Table S2**), which was the basis for the elaboration of results. The nomenclature of vascular plants is given in Mirek et al. (2020) and that of moss in Hodgetts et al. (2020). Protected species were distinguished on the basis of the Regulation of the Minister of Environment (2014) and identified as threatened on the basis of the Red List (Kaźmierczakowa, 2016). Syntaxonomic classification was conducted based on the Matuszkiewicz guide (Matuszkiewicz, 2005) and Ratyńska et al. (2010).

New occurrences were documented according to ATPOL grid square references (Zając, 1978).

### 2.3 Data analysis

Data were analyzed using the STATISTICA 13.3 software (TIBCO Software Inc., Palo Alto, CA, USA) and PAST software (PALSTAT, developers Hammer, Ø., Ryan, PD, and David AT Harper). Analyses were performed on 122 phytosociological relevés. The Braun-Blanquet scale coverage values were transformed to a numerical scale of 1 – 9 (van der Maarel, 1979). The dendrogram was prepared using the unweighted paired group method with arithmetic mean (UP-GMA). It allowed for the inclusion of relevés in the phytosociological table groups homogeneous with respect to the quantitative arrangement of species in the communities.

In addition, the phytosociological relevés were divided into two groups: with the participation of *E. hieracifolia* (group 2) and without (group 1). Differences in species composition between them were checked using a one-way PERMANOVA. To indicate which species played the greatest role in the difference between the two studied groups, the Similarity Percentage analysis (SIMPER) analysis was performed. In the case of both analyses, the Bray–Curtis distance measure was used. The analyses were performed based on the abundance of 24 species, which show loading over 0.1 with the first three axes of principal component analysis (PCA).

Phytosociological relevés with and without *E. hieracifolia* were compared using the Mann–Whitney U test as well as Taxa (S), Simpson (SIMP = 1 − D), and Shannon–Wiener (H′) indices. A comparison was also made showing population dynamics over 2 years in the case of phytosociological relevés 1 – 60 and 1A – 60A. It was based on the quantity of the species.

## 3 Results

### 3.1 Vegetation

All phytosociological relevés together contained 47 species of vascular plants and 10 species of bryophytes (**Supplementary Table S2**). There were two alien species: *E. hieracifolia* and *Padus serotina*. Six species of vascular plants and almost all bryophytes are rare or protected plants. *Drosera rotundifolia* and *Scheuchzeria palustris* are under strict species protection (Regulation of the Minister of Environment, 2014) and are on the Red List (Kaźmierczakowa, 2016) with the categories near threatened (NT) and vulnerable (VU), respectively. *Andromeda polifolia*, *Ledum palustre*, *Menyanthes trifoliata*, and all bryophytes (except *Polytrichum juniperinum*) are under partial species protection (Regulation of the Minister of Environment, 2014). *Rhynchospora alba* is on the Red List (Kaźmierczakowa, 2016).

**Figure 4:**
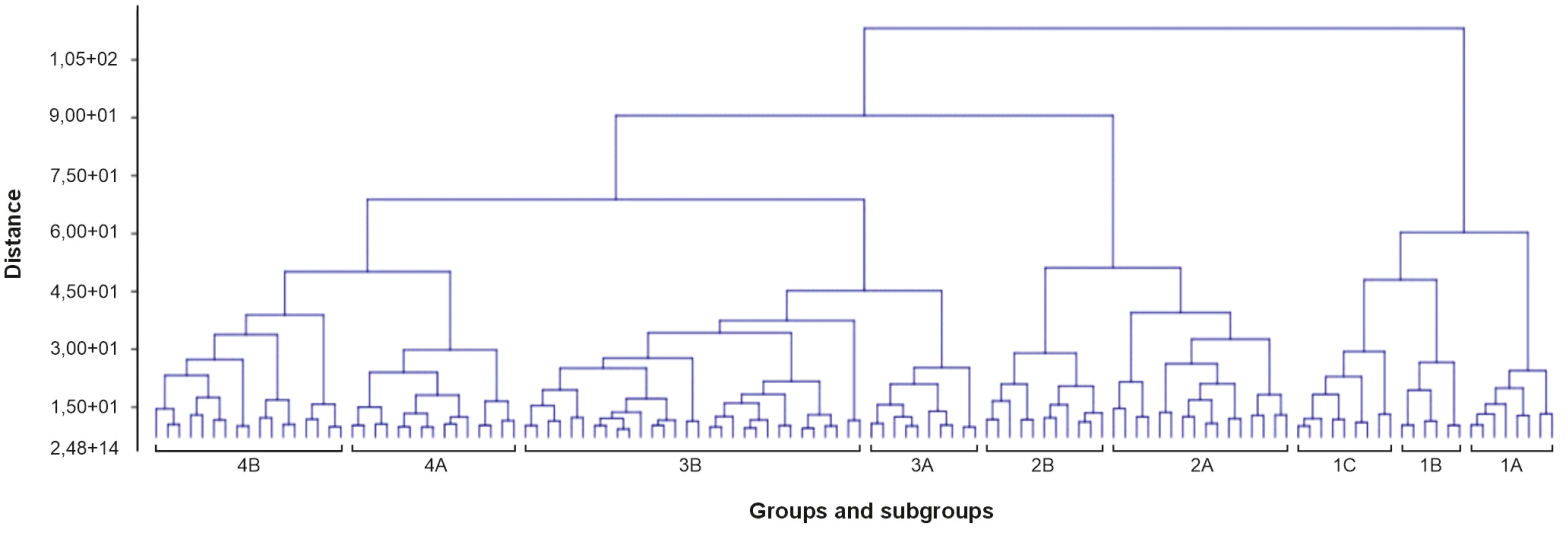
A dendrogram made based on cluster analysis (unweighted paired group method with arithmetic mean and Ward’s clustering method) shows a floristic similarity between the phytosociological relevés, showing four recognized main floristic groups. Numbers (1–62) indicate phytosociological relevés described in **Supplementary Table S1**.

The numerical classification (**Figure 4**) made it possible to organize the phytosociological table (**Supplementary Table S2**) and distinguish the following plant communities that occurred in the analyzed area:

Class: *Oxycocco-Sphagnetea* Br.–Bl. et R. Tx. 1943
Order: *Sphagnetalia magellanici* (Peacockłowski in Pawłowski et al., 1928) Kandsten and
Floßner 1933
Alliance: *Sphagnion magellanici* Caster and Flossner 1933
Association: *Ledo–Sphagnetum magellanici* Sukopp 1959 em. Neuhandusl 1969
Association: *Sphagno recurvi–Eriophoretum vaginati* Hueck 1925 nom. invers
Class: *Scheuchzerio-Caricetea fuscae* (Nordhagen 1936) R.Tx. 1937
Order: *Scheuchzerietalia palustris* Nordhagen 1936
Alliance: *Rhynchosporion albae* W. Koch 1926
Association: *S. recurvi–Eriophoretum angustifolii* Hueck 1925 nom. invers. et
nom. mut.
Order: *Caricetalia fuscae* W. Koch 1926
Alliance: *Caricion fuscae* Koch 1926 em. Klika 1934
Association: *Sphagno–Juncetum effusi* Dziubałkowski 1928
Alliance: *Sphagno–Caricion canescentis* Passage (1964) 1978
Association: *S. recurvi–Caricetum rostratae* Steffen 1931

It was not always possible to identify specific plant communities. Four main floristic groups were recognized (**Figure 4**) based on hierarchical cluster analysis (UP-GMA).

Group 1 included communities from the *Scheuchzerio-Caricetea fuscae* class. All phytosociological relevés contained *Sphagnum fallax*, reaching an average cover of approximately four. The average number of plant species was also high (11.8) in relation to the other groups. Subgroup 1A included the *Sphagno–Juncetum effusi* community. *Juncus effusus* achieved an equally high average cover (apart from *S. fallax*). The shrub layer was also quite compact, built by *Salix aurita* and regenerative shoots of *Betula pubescens*. It was difficult to unequivocally assign the plant community to group 1B. These were phytosociological relevés from patch 7, which was a degraded habitat (7140 Transition mires and quaking bogs), largely overgrown by *Alnus glutinosa*, *B. pubescens*, and *Frangula alnus*. The bryophyte layer occupied significant areas and was mainly formed by several peat moss species, including *S. fallax* and *Sphagnum divinum*. From the *Scheuchzerio-Caricetea fuscae* class, *Carex lasiocarpa*, *Carex nigra*, *S. palustris*, and *Viola palustris* grew. However, they had a small share. Species from the classes *Alnetea glutinosae* and *P. australis* predominated. In the last subgroup, two plant communities could be distinguished, which, for example, in patch 1, created a mosaic. These were the *S. recurvi–E. angustifolii* associations from the *R. albae* alliance and the *S. recurvi–C. rostratae* associations from the *Sphagno–Caricion canescentis* alliance. The first one was characterized by a large share of *Eriophorum angustifolium*, while the second was *Carex rostrata* with a simultaneous large share of *S. fallax*. Other *Sphagnum* species occurred sporadically, but *Aulacomnium palustre* also grew. In some places, *Oxycoccus palustris* also reached significant coverage. In group 1, *E. hieracifolia* was recorded in two phytosociological relevés. In both cases, it was the *S. recurvi–C. rostratae* association in patch 5 (relevés 24A) and on a peat bog in the Bagno Przecławskie reserve. This is also a group where water stagnates at least in the spring.

The next three groups were represented mainly by communities from the class *Oxycocco-Sphagnetea*, although the last one contained phytosociological relevés with species composition more appropriate to the class *Scheuchzerio–Caricetea fuscae*.

Group 2 was characterized by the participation of regenerative shoots of *B. pubescens* and saplings of *Pinus sylvestris*, mainly in the shrub layer (the stand built by these species was mostly removed in 2019). *Eriophorum vaginatum* reached an average degree of cover of four, and *S. fallax* reached three. In most phytosociological relevés, *O. palustris* had a large representation. The participation of other species determined the communities. In subgroup 2A, the *S. recurvi–E. vaginati* community could be distinguished, where both species had a cover of up to 50%–90%. Other *Sphagnum* species rarely grew; *A. palustre* appeared on low covers. Subgroup 2B was represented by the *Ledo-Sphagnetum magellanici* community, although there was most likely an initial phase within the *E. vaginatum–S. fallax* association. *S. fallax* still had a large share, while *S. divinum*, for example, achieved small coverage. *L. palustre*, as a typical species, covered 25% to 60% of the area of phytosociological relevés. Saplings of *P. sylvestris* also appeared. Group 2 was characterized by a high average number of plant species (11.9), as well as a high average number of bryophyte species (3.4). Also in this group, *E. hieracifolia* appeared sporadically (phytosociological relevés 14, 19A, 21, and 21A).

Group 3 was distinguished by the low coverage of the shrub layer (mainly regenerative shoots of *B. pubescens*, as well as *F. alnus*) and the share of *M. caerulea* in the ground cover, growing between *E. vaginatum* clumps, which, together with *S. fallax*, built the *S. recurvi–E. vaginati* association. This group was also characterized by the occurrence of *E. hieracifolia*. It appeared in almost all phytosociological relevés, sometimes reaching up to 20% coverage. Two subgroups could be distinguished. Subgroup 3A was characterized by the share of *M. caerulea* at an average level of coverage of approximately 3, while in subgroup 3B, this share was lower (approximately 1 average degree of coverage). The average number of species was the lowest here (9.6).

Group 4 was represented by the heterogeneous *Oxycocco-Sphagnetea* and *Scheuchzerio-Caricetea fuscae* classes. Most likely, such a distribution was determined by the smallest share of the shrub layer among all groups. Although *B. pubescens* (regenerative shoots of) appeared in most of the phytosociological relevés, these were usually low covers, not exceeding 5% or even 1%. Also, the fact that in some patches the communities formed mosaics could have determined such an assignment. *E. vaginatum* and *S. fallax* stood out with a significant share. The *S. recurvi–E. vaginati* and *Sphagno–Juncetum effusi* associations could be distinguished. Subgroup 4A consisted mainly of phytosociological relevés taken within the first association. It was distinguished by a large share of *C. lasiocarpa*, and in the case where *E. vaginatum* achieved high levels of cover, *C. lasiocarpa* achieved low levels, and vice versa. *Calla palustris* was found growing in the depressions among *E. vaginatum*. Group 4B largely represented the *Sphagno–Juncetum effusi* association with dominant *J. effusus* (except phytosociological relevés 25, 25A, 27, and 27A). *E. hieracifolia* appeared in most phytosociological relevés, but achieved significant coverage.

### 3.2 Analysis of differences in peatland patches with and without *E. hieracifolia*

**Figure 5:**
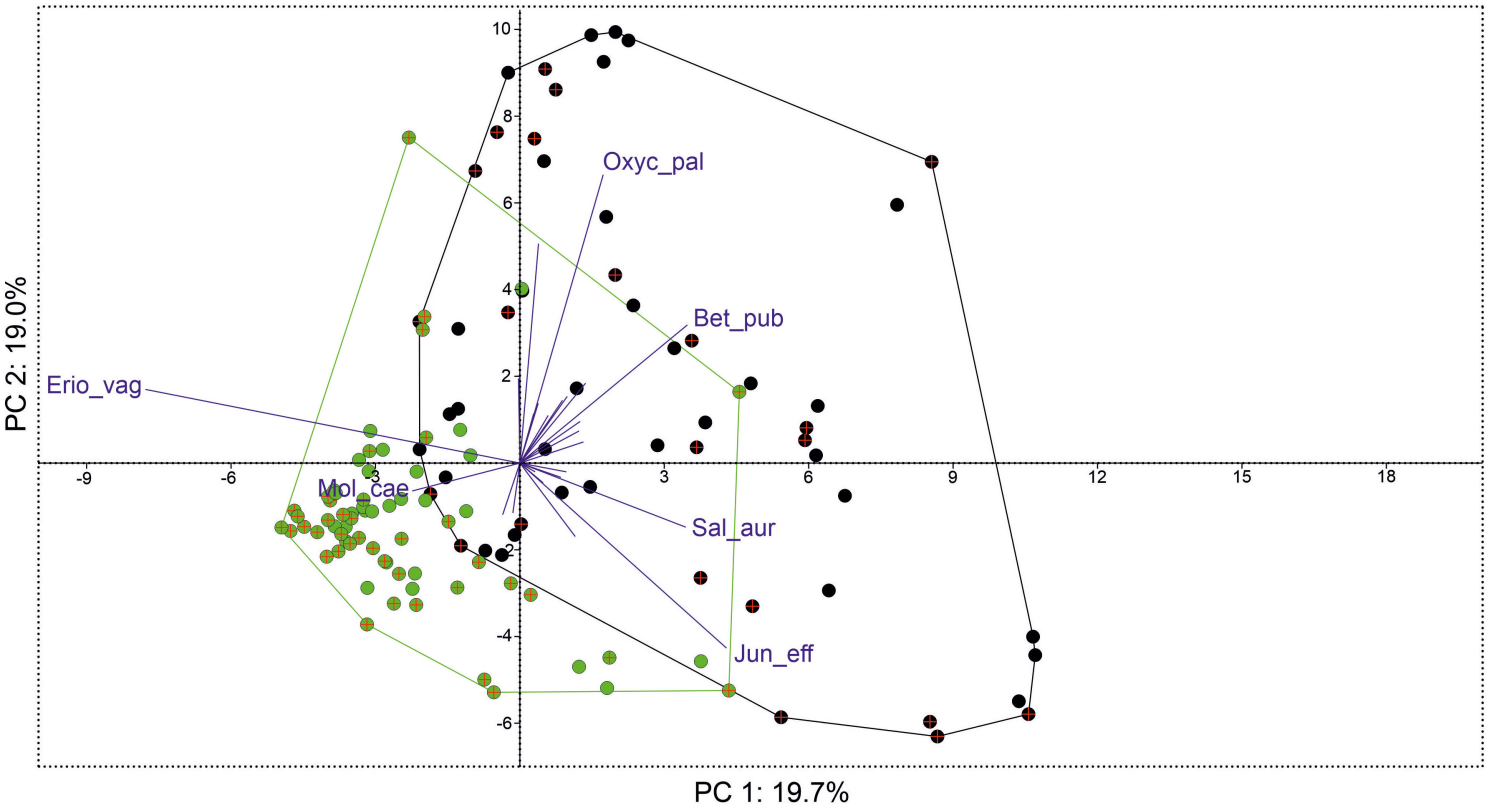
Results of principal component analysis (PCA) showing scatterplot of phytosociological relevés with the share of *Erechtites hieracifolia* (2019, green points; 2022, green point with the cross) and without the share (2019, black points; 2019, black points with the cross) with biplot of species contribution (only species with loadings > 0.1 with the first three components were used).

PERMANOVA showed significant differences in species coverage between groups with and without the share of *E. hieracifolia* (F = 15.02, p = 0.0001). Comparing species abundance, overall dissimilarities between the studied groups were 58.65%. *E. vaginatum*, *B. pubescens* (layer A), and *O. palustris* showed the highest contribution to the dissimilarity between studied groups (together over 20%). *E. vaginatum* had a significantly larger share in the plots with *E. hieracifolia*, and the other two species were more abundant in the plots without *E. hieracifolia* (**Table 2**). Somehow similar results were shown by PCA, with two slightly overlapping groups of points (plots with and without share of *E. hieracifolia*) (**Figure 5**). The first three axes together explained 50.3%. The studied groups were separated along both the first and second axes, with *E. vaginatum* and *E. hieracifolia* highly correlated with the first axis, and *O. palustris* and *L. palustre* with the second axis. The full results of the analysis are presented in **Supplementary Table S3**.

**Table 2:** Results of SIMPER analysis.

Species	Average dissimilarity	Contribution (%)	Cumulative contribution (%)	Mean abundant Group 1	Mean abundant Group 2
*Eriophorum vaginatum*	4.34	7.41	7.41	4.98	7.63
*Betula pubescens*	3.96	6.75	14.16	3.42	0.75
*Oxycoccus palustris*	3.46	5.91	20.06	2.82	0.55
*Juncus effusus*	2.96	5.06	25.12	1.85	1.10
*Betula pendula_*b	2.62	4.47	29.59	2.60	2.73
*Molinia caerulea*	2.46	4.19	33.79	0.64	1.78
*Carex lasiocarpa*	2.82	3.89	37.68	1.16	1.36
*Frangula alnus*	2.07	3.53	41.21	1.42	1.22
*Ledum palustre*	2.00	3.41	44.62	1.60	0.22
*Pinus sylvestris*_b	1.99	3.39	48.01	1.49	0.76
*Carex rostrata*	1.73	2.94	50.95	0.89	0.60
*Salix aurita*	1.63	2.78	53.73	1.27	0.06
*Sphagnum fallax*	1.60	2.73	56.46	7.18	7.09
*Pinus sylvestris_c*	1.59	2.71	59.17	1.25	0.19
*Aulacomnium palustre*	1.50	2.56	64.32	1.27	0.84
*Sphagnum divinum*	1.52	2.59	61.76	1.29	0.15
*Calamagrostis canescens*	1.45	2.48	66.80	0.71	0.70
*Sphagnum palustre*	1.41	2.41	69.21	1.11	0.22
*Eriophorum angustifolium*	1.23	2.11	71.31	0.71	0.31
*Calla palustris*	1.18	2.02	73.33	0.26	0.79

Contribution to the dissimilarity between the studied groups [without *Erechtites hieracifolia* (group 1), and with the share of *E. hieracifolia* (group 2)].

Indices calculated showed significant differences in species coverage between studied groups (with and without the share of *E. hieracifolia*) (**Table 3**). The total number of species and the mean number of species in the group with *E. hieracifolia* were lower than those in the group without it. Based on the Shannon–Wiener diversity index (H′), plant species diversity in both groups was quite high. Interestingly, the Shannon–Weiner index was higher in the group without *E. hieracifolia*, so this group had greater diversity. Similarly, the Simpson index diversity (1 − D) reached lower values in the communities with *E. hieracifolia*, indicating lower diversity in these samples.

**Table 3:** The total number of species and mean (range) ± SD of the number of species in a phytosociological relevé (S), as well as Shannon–Wiener (H′) and Simpson indices of diversity (as its complement SIMP = 1 − D) calculated in two plant communities with and without *Erechtites hieracifolia*.

Groups	1 Without *E. hieracifolia*	2 With *E. hieracifolia*	The Mann–Whitney U test; p-value
Total number of species	20	13	–
S	11.65 ± 3.07	9.87 ± 2.00	1,156.50; 0.00420
H′	2.29 ± 0.28	2.11 ± 0.21	1,112.50; 0.00017
SIMP	0.88 ± 0.04	0.86 ± 0.03	1,042.50; 0.00004

### 3.3 Changes in *E. hieracifolia* coverage

**Figure 6:**
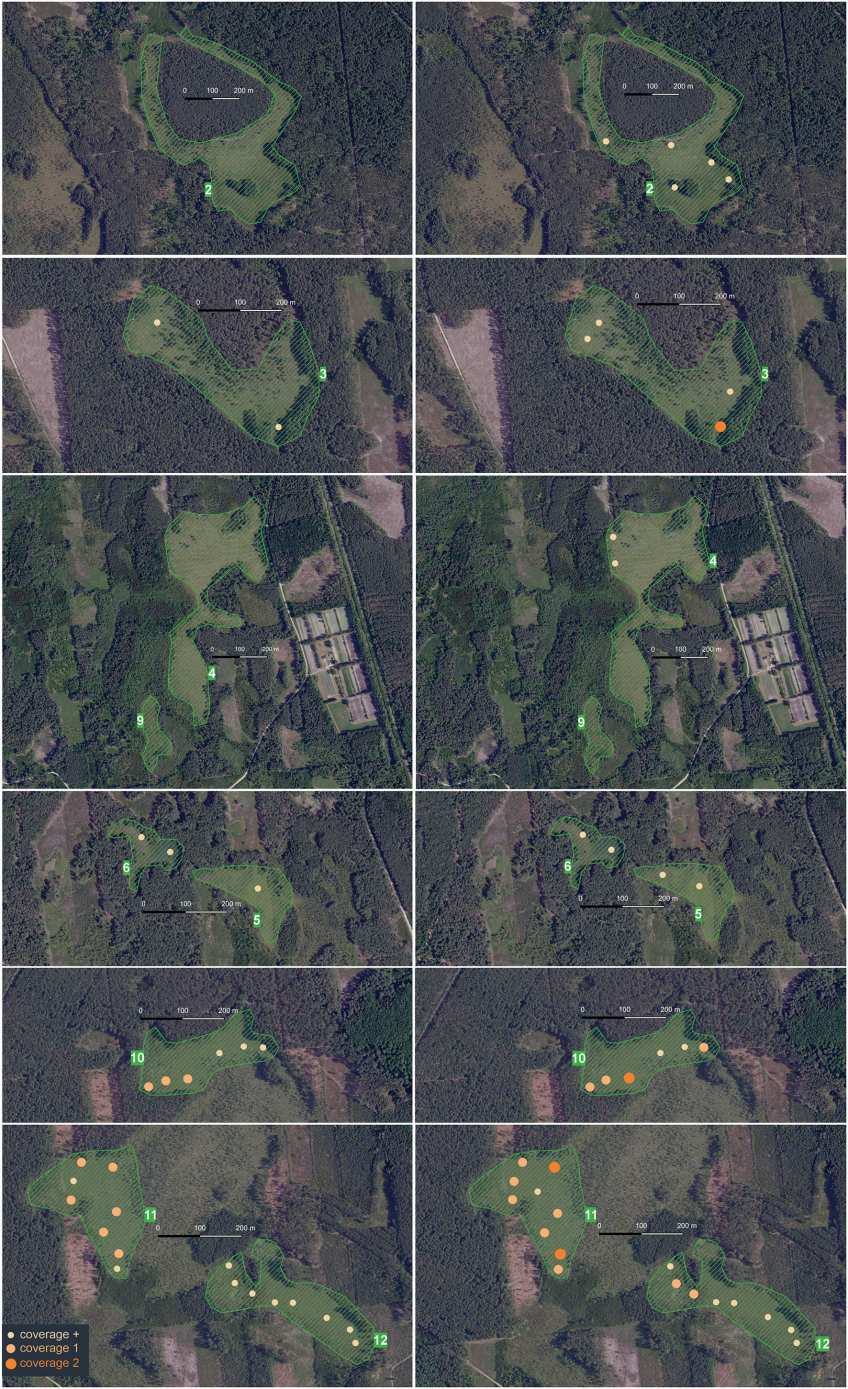
Spread of *Erechtites hieracifolia* in 2019–2022 in the patches with the species (own work based on the map from the website Geoportal 2).

In 2019, the species occurred in six of the studied peatland patches (3, 5, 6, and 10 – 12), while in 2020, the species occurred in eight (2 – 6 and 10–12). In patches 2 and 4, the species did not occur at all in 2019, while in all the others, it increased its cover. The greatest changes were observed in patch 11 (**Figure 6**).

Comparison of the coverage of *E. hieracifolia* (expressed on the van der Maarel scale) in individual phytosociological relevés in 2019 and 2022 showed that in 18 relevés, its coverage remained at a constant level; in nine, it increased; and in 12, the species was recorded in 2022 for the first time.

### 3.4 New localities of *E. hieracifolia* for Poland

The studied *E. hieracifolia* sites are also new sites of the species in Poland. The peatland patches are located within two ATPOL cartogram squares: GE04 and GE05 (**Figure 2-right**). Furthermore, new species sites were found in squares EF69 and FF40, where 61 and 62 phytosociological relevés were conducted, respectively (**Table 1**).

## 4 Discussion

Wetlands, including peatlands, are among the ecosystems with the lowest levels of colonization by alien plant species in Europe. Nevertheless, they are considered among the most threatened habitat types. The main factors of their degradation are intensive anthropogenic changes, such as drainage, as well as climate change. These factors reduce ecological stability, thereby increasing the vulnerability of these ecosystems to biological invasions.

In recent years, one of the species that has started to appear in such habitats is the studied species *E. hieracifolia* (e.g., Krawczyk, 2010; Orlov and Iakushenko, 2011; Koczywąs et al., 2012; Dyderski et al., 2015), which had previously been associated mainly with heavily human-altered environments such as roadsides, clear-cuts, and post-fire areas (e.g., Górski et al., 2003; Dvořáková, 2004). A similar pattern of expansion was observed in the study area. For several years, the species spread mainly in clear-cut areas, forming populations of several hundred individuals, before gradually encroaching into adjacent peatland ecosystems. Wind likely played a key role in the spread of *E. hieracifolia*, particularly in patches located near already colonized areas. For instance, patch 11—hosting the largest population within the peatland area—was situated just several dozen meters from a clear-cut where the species occurred in the thousands, making it highly susceptible to colonization by wind-dispersed seeds. Although our study focused primarily on floristic composition and site characteristics, we acknowledge that the spatial configuration of the landscape significantly influences the species’ invasion dynamics, especially in small exposed patches adjacent to disturbed areas. Additionally, in 2019/2020, woody vegetation was cleared on survey patches. There is a high probability that seeds were also transported by vehicles used for felling trees (Górski et al., 2003; Von der Lippe and Kowarik, 2007; Bohdan and Sulej, 2020; Zaniewski et al., 2020). Despite the typically forested surroundings of the study peatland patches, the landscape is intersected by a dense network of access roads (**Figure 3**), which could have facilitated seed dispersal via forestry vehicles.

Peatlands colonized by *E. hieracifolia* are most often degraded (e.g., Orlov and Iakushenko, 2011; Zaniewski et al., 2020). A similar situation was observed in the study area. Most of the peatland patches in our study with this species could be classified as raised bogs, corresponding to natural habitat types 7110 and 7120 (**Table 1**, **Supplementary Table S1**), but many of them were ecologically degraded. The most significant factor contributing to the unfavorable conservation status was drainage and drought, which led to a lowering of the groundwater level. In well−preserved raised bogs, the groundwater table is expected to remain consistently high (Stańko, 2010), although in continental−type raised bogs, the water table may experience significant intra−annual fluctuations (Wysocki and Sikorski, 2014). Nevertheless, the patches colonized by *E. hieracifolia* in the studied area were characterized by groundwater fluctuations not only within individual years but also between years. In 2019, no groundwater was observed even at 10 cm below the peat surface in many of these sites (even in spring) (e.g., patches 2 – 4), while in 2022, water persisted above ground level, accumulating in hollows between tussocks of *E. vaginatum*. Such conditions may facilitate the expansion of *E. hieracifolia*, a species that is intolerant to high groundwater levels (Darbyshire et al., 2012). In contrast, patch 1, a transitional mire surrounded by pine and swamp forests, maintained good water conditions during both study years, and *E. hieracifolia* was not recorded there.

Hydrological fluctuations, combined with both low *Sphagnum* moss cover and low *Sphagnum* species richness, indicate a disruption in peatland functioning. Peat-forming bryophytes occur mainly as scattered tufts and do not form a continuous, active acrotelm layer. These structural conditions indicate peat accumulation and progressive habitat degradation resulting from a breakdown in natural peat-forming processes (Zając et al., 2018). The presence of *E. vaginatum* with very high cover (70%–90%)—a species recognized as an indicator of degraded peatlands (Kujawa–Pawlaczyk and Pawlaczyk, 2014)—further supports the diagnosis. Given the above and the fact that *E. hieracifolia* was most frequently recorded in the *S. recurvi–E. vaginati* plant community (the *Oxycocco-Sphagnetea* class), it can be assumed that *E. vaginatum* may potentially play an important role in the colonization of peatlands by *E. hieracifolia*. Most frequently, *E. hieracifolia* was observed growing directly on the tussocks formed by *E. vaginatum* (**Figure 1**). We suspect that *E. vaginatum* may promote the colonization of *E. hieracifolia* by providing supportive microclimatic conditions (Lavoie et al., 2003). PCA also confirmed that *E. vaginatum* had a significantly higher share in the group with *E. hieracifolia* (**Table 2**, **Figure 5**). Similar conclusions can be drawn from the dendrogram (**Figure 4**), where *E. hieracifolia* showed significant presence in groups 3 and 4, also characterized by high *E. vaginatum* cover. This dependence was especially visible in patches 2, 3, and 11, where *E. vaginatum* formed dense tussocks sparsely interspersed with *Sphagnum*, creating suitable microhabitats for colonization. In contrast, the mosaic of *C. rostrata* and *S. fallax* observed in patch 1 seemed less favorable. Similarly, although *E. hieracifolia* was recorded by Dyderski et al. (2015) in the *S. recurvi–E. angustifolii* plant community (class *Scheuchzerio-Caricetea fuscae*), it is worth noting that *E. vaginatum* was also present in that community. Likewise, observations were made in a well-hydrated peatland in the Bagno Przecławskie reserve, where *E. hieracifolia* was found growing on tussocks of *E. vaginatum*, which occurred as an admixture within plant communities typical of transitional mires.

The calculated values of the Shannon–Wiener and Simpson indices (D – 1) in our study were relatively high. In comparison, lower index values (1.29 – 1.53) were reported in plant communities of lake and river mires in SE Poland (Sugier and Czarnecka, 2012) and the Łęczna–Włodawa Lake District (Czarnecka and Franczak, 2015), but in these studies, the indices were calculated separately for individual associations. In our case, indices were calculated for groups with and without *E. hieracifolia*. Each group included different vegetation types, which may explain the relatively high overall diversity. At the same time, the value of the Shannon–Weiner index calculated in our study was slightly lower than the value recorded in selected mires on the eastern shore of the Gulf of Bothnia (Finland) (Laine et al., 2018). Those Finnish peatlands were undisturbed and at a natural successional stage, characterized by higher microhabitat heterogeneity, which likely contributed to the higher species richness. Diversity indices and PERMANOVA results indicated significant differences in species composition between groups with and without the presence of *E. hieracifolia*. The Shannon–Wiener index for the group with *E. hieracifolia* was slightly lower, suggesting lower species richness. A similar pattern was observed for the Simpson index (1 − D), with lower values in the group colonized by *E. hieracifolia*, suggesting reduced floristic diversity. All results suggest that *E. hieracifolia* preferentially colonizes habitats where biodiversity has already declined, most likely as a consequence of hydrological disturbances such as desiccation. Similar observations have been reported by Liu et al. (2024), who noted a decrease in the Shannon–Wiener index values in response to decreasing water levels. Pioneer species like *E. hieracifolia* are generally associated with degraded habitats characterized by simplified floristic structure (e.g., Dyderski et al., 2015; Zaniewski et al., 2020). Indeed, most plant communities in which the species was recorded in our study area had a simplified floristic structure. In the majority of these patches, *E. vaginatum* was the dominant species, forming tussocks only sparsely covered by *S. fallax*. The total *Sphagnum* cover often did not exceed 10%. These characteristics are indicators of poor habitat condition (Stańko, 2010). The degradation of habitat conditions, including lowered groundwater levels, can reduce the competitiveness of native peatland species, facilitating the invasion of alien taxa adapted to environmental stress (Minayeva and Sirin, 2012; Tanneberger et al., 2021), such as *E. hieracifolia*. It cannot, therefore, be ruled out that the increasing abundance of *E. hieracifolia* may further accelerate the decline in biodiversity, which is particularly concerning in the case of peatlands, which are extremely sensitive and ecologically valuable habitats (Koczywąs et al., 2012). They provide habitats for specialized species adapted to life in wet, acidic, and nutrient-poor habitats (Assessment on Peatlands, Biodiversity and Climate Change, 2008; Sender et al., 2022).

The studied species preferred moderately exposed sites. Most often, it occupied the edges of peatlands bordering forests, shading the habitat, at least at a certain time of day, or near trees or shrubs. Trotta et al. (2023) and Koczywąs et al. (2012) indicated similar habitat conditions to the occurrence of this species. In turn, Zaniewski et al. (2020) confirmed that this species has become more shade-tolerant than in its main range in Poland.

The results of our research and direct field observations indicate that *E. hieracifolia* is expanding its range in the studied peatland patches (e.g., patches 3 and 6). It has also appeared in previously uncolonized patches (e.g., patches 2 and 4) (**Figure 6**, **Supplementary Table S2**). The size of the species population in one of the most degraded patches (11) is concerning, with *E. hieracifolia* covering almost 25% of the phytosociological relevé (**Figure 1**). Orlov and Iakushenko (2011) reported a population occupying 4 ha in a drained peatland, so we agree that this species may be aggressive in peatlands (e.g., Cameron and de Lange, 2002; Trotta et al., 2023). It is likely that *E. hieracifolia* may soon spread to structurally similar peatland patches in the study area. The risk is heightened by projected climate change, which may result in more frequent droughts or wildfires (Assessment on Peatlands, Biodiversity and Climate Change, 2008), exacerbating mire degradation and increasing their susceptibility to invasion.

Although studies have indicated that this species is a weak competitor and that its populations may decline over time (Peterson and Pickett, 1995; Wołkowycki and Wołkowycki, 2023), this trend is uncertain in open peatland habitats, where competitive pressure remains relatively stable and ongoing active protection is in place. Therefore, ongoing monitoring of the *E. hieracifolia* population is important, especially when it occurs in natural habitats of high conservation value. In sites of exceptional ecological importance, especially where populations are still small, early eradication efforts may be warranted. Preventive measures may help limit further spread. Although eradication may be difficult due to the species’ persistent seed bank (Baskin and Baskin, 1996), which can be activated by soil disturbance or fire (Celka et al., 2017), early intervention may limit long-term impacts. According to Tichý et al. (2023), *E. hieracifolia* prefers habitats with low to moderate moisture levels. This suggests that maintaining consistently high groundwater levels could limit its establishment. However, under current climatic conditions—characterized by recurring annual droughts—the implementation of such hydrological measures appears unlikely, especially in raised bogs, which are already severely drained. Some benefits could potentially be achieved through local interventions, such as the removal of drainage ditches (a remnant of historical land-use practices), although their effectiveness in the current climate would likely be limited. This species is known to be sensitive to frequent, low-intensity mowing, which can be a viable control method in open habitats. In the case of small populations that are sparse, manual removal may also be effective. In degraded or low-conservation-value sites, herbicide application could be considered as a last resort (Atkinson et al., 2014). Importantly, eradication should be prioritized in areas where *E. hieracifolia* forms large populations—such as clear-cuts—which serve as focal points for further spread.

New occurrences of *E. hieracifolia* are continuously being reported not only in Poland but also across other parts of Europe, indicating the ongoing expansion of this species. Given the open nature of peatland habitats and the species’ ability to persist under stable competition conditions, further research is necessary to assess long-term population dynamics and environmental impact. Future studies should consider detailed environmental parameters—such as hydrological regime—along with population structure and cover. In addition, spatial analysis—including landscape connectivity, distance to source population, and dominant wind direction—will be essential for understanding dispersal mechanisms and invasion risk. Expanding research to a broader geographical range and into similar habitat types will be crucial for understanding the potential threat posed by *E. hieracifolia* and for developing appropriate conservation or eradication strategies. Due to the lack of confirmed data, it also seems justified to investigate whether *E. hieracifolia* exhibits allelopathic effects, a mechanism that has not yet been documented in the scientific literature.

## 5 Conclusions

In southeastern Poland, *E. hieracifolia* is constantly expanding its range, inhibiting degraded peatlands. It most frequently grows in the *S. recurvi–E. vaginati* community. Over the course of 3 years, it has increased its range within the studied patches, or it appeared in the next patches. In the study area, the group of communities with the recorded presence of *E. hieracifolia* was characterized by lower species diversity, expressed by the average species in the phytosociological relevé, and higher diversity expressed by the H′ and SIMP indices. The groups differed significantly, as confirmed by PCAs. *E. hieracifolia* colonizes habitats that have already experienced a decline in diversity, most likely as a consequence of hydrological disturbances such as desiccation. Additionally, it most often inhabits patches with a large proportion of *E. vaginatum*. Possibly, the deepening drought and the increase in the species population may even more threaten the biodiversity of the occupied habitats. Further research on the dynamics of the species population and continuous monitoring are necessary, especially in protected areas. Elimination of the species from the places most exposed to its penetration should also be considered.

## Data Availability

The original contributions presented in the study are included in the article/Supplementary Material. Further inquiries can be directed to the corresponding author.
