# Supplementary material for: Erechtites hieracifolia: an invasive plant species in peatland habitats of southeastern Poland (Central Europe)
Source: Front Plant Sci. 2025 Sep 10;16:1615073. doi: 10.3389/fpls.2025.1615073 (PMC12457311; doi:10.3389/fpls.2025.1615073)
Supplement: Supplementary file 1 [file Table1.docx]

Tabela S2. Results of SIMPER analysis

| **Taxon** | **Average dissimilarity** | **Contribution [%]** | **Cumulative contribution [%]** | **Mean abundant Group 1** | **Mean abundant Group 2** |
| --- | --- | --- | --- | --- | --- |
| *Eriophorum vaginatum* | 4.34 | 7.41 | 7.41 | 4.98 | 7.63 |
| *Betula pubescens* | 3.96 | 6.75 | 14.16 | 3.42 | 0.75 |
| *Oxycoccus palustris* | 3.46 | 5.91 | 20.06 | 2.82 | 0.55 |
| *Juncus effusus* | 2.97 | 5.06 | 25.12 | 1.85 | 1.10 |
| *Betula pubescens_b* | 2.62 | 4.47 | 29.59 | 2.60 | 2.73 |
| *Molinia caerulea* | 2.46 | 4.19 | 33.79 | 0.64 | 1.78 |
| *Carex lasiocarpa* | 2.28 | 3.89 | 37.68 | 1.16 | 1.36 |
| *Frangula alnus* | 2.07 | 3.53 | 41.21 | 1.42 | 1.22 |
| *Ledum palustre* | 2.00 | 3.41 | 44.62 | 1.60 | 0.22 |
| *Pinus sylvestris_b* | 1.99 | 3.39 | 48.01 | 1.49 | 0.76 |
| *Carex rostrata* | 1.73 | 2.94 | 50.95 | 0.89 | 0.60 |
| *Salix aurita* | 1.63 | 2.78 | 53.73 | 1.27 | 0.06 |
| *Sphagnum fallax* | 1.60 | 2.73 | 56.46 | 7.18 | 7.09 |
| *Pinus sylvestris* | 1.59 | 2.71 | 59.17 | 1.25 | 0.19 |
| *Sphagnum divinum* | 1.52 | 2.59 | 61.76 | 1.29 | 0.15 |
| *Aulacomnium palustre* | 1.50 | 2.56 | 64.32 | 1.27 | 0.84 |
| *Calamagrostis canescens* | 1.45 | 2.48 | 66.80 | 0.71 | 0.70 |
| *Sphagnum fallax* | 1.41 | 2.41 | 69.21 | 1.11 | 0.22 |
| *Eriophorum angustifolium* | 1.23 | 2.11 | 71.31 | 0.71 | 0.31 |
| *Calla palustris* | 1.18 | 2.02 | 73.33 | 0.26 | 0.79 |
| *Betula pubescens_c* | 1.11 | 1.89 | 75.22 | 0.33 | 0.79 |
| *Vaccinium myrtillus* | 1.01 | 1.71 | 76.93 | 0.73 | 0.27 |
| *Lysimachia thyrsiflora* | 0.90 | 1.54 | 78.47 | 0.55 | 0.31 |
| *Phragmites australis* | 0.90 | 1.53 | 80.01 | 0.36 | 0.49 |
| *Lysimachia vulgaris* | 0.87 | 1.48 | 81.49 | 0.42 | 0.43 |
| *Dryopteris cristata* | 0.83 | 1.42 | 82.91 | 0.36 | 0.48 |
| *Carex nigra* | 0.82 | 1.39 | 84.30 | 0.40 | 0.40 |
| *Pleurozium schreberi* | 0.80 | 1.37 | 85.67 | 0.36 | 0.42 |
| *Andromeda polifolia* | 0.79 | 1.35 | 87.02 | 0.62 | 0.07 |
| *Vaccinium uliginosum* | 0.75 | 1.28 | 88.30 | 0.62 | 0.03 |
| *Pinus sylvestris* | 0.72 | 1.23 | 89.53 | 0.42 | 0.28 |
| *Alnus glutinosa* | 0.66 | 1.12 | 90.65 | 0.62 | 0.00 |
| *Polytrichum strictum* | 0.59 | 1.01 | 91.66 | 0.44 | 0.09 |
| *Peucedanum palustre* | 0.57 | 0.97 | 92.63 | 0.33 | 0.22 |
| *Carex elata* | 0.39 | 0.67 | 93.30 | 0.07 | 0.27 |
| *Polytrichum juniperinum* | 0.37 | 0.62 | 93.92 | 0.11 | 0.21 |
| *Sphagnum papillosum* | 0.36 | 0.61 | 94.54 | 0.33 | 0.00 |
| *Thelypteris palustris* | 0.35 | 0.59 | 95.13 | 0.07 | 0.21 |
| *Sphagnum capilifolium* | 0.30 | 0.52 | 95.65 | 0.29 | 0.00 |
| *Drosera rotundifolia* | 0.29 | 0.50 | 96.14 | 0.22 | 0.00 |
| *Frangula alnus_c* | 0.26 | 0.45 | 96.59 | 0.04 | 0.18 |
| *Salix cinerea* | 0.25 | 0.42 | 97.01 | 0.11 | 0.10 |
| *Epilobium palustre* | 0.18 | 0.31 | 97.32 | 0.04 | 0.12 |
| *Comarum palustre* | 0.16 | 0.27 | 97.59 | 0.09 | 0.06 |
| *Calamagrostis epigejos* | 0.16 | 0.27 | 97.86 | 0.00 | 0.12 |
| *Selinum carvifolia* | 0.15 | 0.25 | 98.11 | 0.11 | 0.00 |
| *Rubus hirtus* | 0.14 | 0.24 | 98.35 | 0.15 | 0.00 |
| *Menyanthes trifoliata* | 0.10 | 0.18 | 98.53 | 0.09 | 0.00 |
| *Quercus robur* | 0.10 | 0.17 | 98.70 | 0.07 | 0.00 |
| *Polytrichum commune* | 0.09 | 0.15 | 98.85 | 0.09 | 0.00 |
| *Vaccinium uliginosum* | 0.08 | 0.14 | 98.99 | 0.00 | 0.06 |
| *Sambucus nigra* | 0.08 | 0.13 | 99.12 | 0.07 | 0.00 |
| *Scheuchzeria palustris* | 0.08 | 0.13 | 99.25 | 0.07 | 0.00 |
| *Galium palustre* | 0.07 | 0.12 | 99.37 | 0.07 | 0.00 |
| *Equisetum fluviatile* | 0.07 | 0.12 | 99.50 | 0.07 | 0.00 |
| *Viola palustris* | 0.07 | 0.12 | 99.62 | 0.07 | 0.00 |
| *Lycopus europaeus* | 0.07 | 0.12 | 99.74 | 0.07 | 0.00 |
| *Rhynchospora laba* | 0.04 | 0.07 | 99.81 | 0.00 | 0.03 |
| *Carex canescens* | 0.04 | 0.07 | 99.88 | 0.00 | 0.03 |
| *Padus serotina* | 0.04 | 0.06 | 99.94 | 0.00 | 0.03 |
| *Lytrum salicaria* | 0.04 | 0.06 | 100.00 | 0.04 | 0.00 |
