# Supplementary material for: Erechtites hieracifolia: an invasive plant species in peatland habitats of southeastern Poland (Central Europe)
Source: Front Plant Sci. 2025 Sep 10;16:1615073. doi: 10.3389/fpls.2025.1615073 (PMC12457311; doi:10.3389/fpls.2025.1615073)
Supplement: Supplementary file 2 [file Table2.docx]

Table S1. Description of the patches

**Patch 1** is a transitional mire in a successional stage associated with the overgrowing of a water body, with patches of open water surface, mainly surrounded by pine forests, including a swamp forest on the northwestern side. The mosaic consists of communities from the *Scheuchzerio-Caricetea* class, primarily *Sphagno recurvi–Eriophoretum angustifolii* and *Sphagno recurvi–Caricetum rostratae*. The dominant species are *Carex rostrata* and *Sphagnum fallax*, with *Eriophorum angustifolium* occurring at lower cover. *Drosera rotundifolia* is also present at this site. On slightly elevated margins, *Sphagno recurvi–Eriophoretum vaginati* occurs. Water conditions were adequate in both study years.

**Patch 2** is the largest in terms of area among all analyzed Patchs. It is isolated and located within pine forests. Its southeastern part contains a more extensive fragment of a peat bog, whereas in the northwestern direction, its structure becomes more fragmented, with the community forming narrow strips between swamp forest and fresh pine forest. The dominant community is *Sphagno recurvi–Eriophoretum vaginati*, with *Eriophorum vaginatum* and *Sphagnum fallax* as the dominant species. In the drier parts, *Molinia caerulea* appears, while in areas where the peat bog borders the swamp forest, a narrow strip of *Ledo–Sphagnetum magellanici* occurs, with a significant share of *Ledum palustre* and clumps of *Sphagnum magellanicum*. In 2019, the water level was very low, and water was not visible on the surface of the peat bog, even between the clumps of *Eriophorum vaginatum*. In 2022, water stagnated in some places, reaching 10 cm on the surface of the peat bog.

**Patch 3** is located within a pine forest. The dominant community is *Sphagno recurvi–Eriophoretum vaginati*, with *Eriophorum vaginatum* and *Sphagnum fallax* as the dominant species. *Molinia caerulea* has a relatively high cover in some areas, especially in drier parts. In 2019, the water level was very low and not visible on the surface of the peat bog, even between the *Eriophorum vaginatum* clumps. In 2022, water stagnated in some places, reaching 5 cm on the surface of the peat bog.

**Patch 4** has a large area and is adjacent mainly to alder forests, as well as to pine and mixed forests with disturbed species composition. The dominant community is *Sphagno recurvi–Eriophoretum vaginati*, with *Eriophorum vaginatum* and *Sphagnum fallax* as the dominant species. In wetter areas, especially on the margins, small fragments of *Sphagno–Juncetum effusi* occur. In 2019, the water level was more than 30 cm below the peat bog surface, whereas in the current year, water was found below 5 cm in some places and at the surface level in others. The most waterlogged fragment was in the southern part of the Patch.

**Patchs 5 and 6** are relatively small in size. They are surrounded by fragmented pine forests, including degraded swamp forests. In Patch 5, *Sphagno recurvi–Eriophoretum vaginati*, dominated by *Eriophorum vaginatum* and *Sphagnum fallax*, prevails in the eastern part. In the western part, a mosaic of *Sphagno recurvi–Eriophoretum angustifolii* and *Sphagno recurvi–Caricetum rostratae* is overgrown by *Phragmites australis*. In Patch 6, a similar pattern occurs, but *Phragmites australis* has a significantly larger share. Water conditions were very good in both Patchs in both study years, with water level at ground level.

**Patch 7** is classified as habitat type 7140. It is located among deciduous and mixed pine forests. It is an intensively overgrowing Patch with a disturbed species composition, and the community is difficult to define. *Sphagnum fallax* dominates, but *Sphagnum divinum* also has a considerable share in some areas. The Patch is further overgrown by *Phragmites australis*. In both 2019 and the current year, the water level was 10–20 cm below the peat bog surface.

**Patch 8** is relatively large and relatively isolated. It is surrounded by pine forests, mainly fresh and mixed, with poorly developed swamp forests also present. Despite its size, the Patch is relatively homogeneous, forming a mosaic of *Sphagno recurvi–Eriophoretum vaginati* and *Ledo–Sphagnetum magellanici*. In 2019, the water level was more than 30 cm below the peat bog surface, whereas in the current year, water was at the peat bog surface in some places.

**Patch 9** is small in size and located among willow thickets and pine forests. The dominant community is *Sphagno–Juncetum effusi*, but advanced succession of willows, mainly *Salix aurita*, has been observed. In 2019, the groundwater level was low and not visible, whereas in 2022, water conditions varied, with water reaching the surface in some places.

**Patch 10** is of medium size and is surrounded mainly by fresh pine forests and mixed stands. The dominant community is *Sphagno recurvi–Eriophoretum vaginati*, while in the northwestern part, patches with a significant share of *Molinia caerulea* appear. Hydrological conditions are variable, with water reaching the surface in some places.

**Patch 11** is of medium size and borders a degraded peat bog fragment as well as pine forests and willow thickets. The dominant community is *Sphagno recurvi–Eriophoretum vaginati*, although the share of peat mosses is relatively low. In some areas, *Molinia caerulea* has a significant presence. In 2019, the water level was more than 30 cm below the peat bog surface. In the current year, hydrological conditions have changed, with water reaching up to 30 cm above the surface in some places, indicating increased waterlogging.

**Patch 12** is of medium size and, like Patch 11, is adjacent to a degraded peat bog fragment as well as pine forests and willow thickets. The dominant community is *Sphagno recurvi–Eriophoretum vaginati*, although the share of peat mosses is relatively low. *Molinia caerulea* has a significant presence in some areas. In the western part of the Patch, a small fragment of *Sphagno–Juncetum effusi* occurs. In some places, the water level reaches the surface of the peat bog.
